# Food Safety Considerations Related to the Consumption and Handling of Game Meat in North America

**DOI:** 10.3390/vetsci7040188

**Published:** 2020-11-25

**Authors:** Hayden D. Hedman, Csaba Varga, Jared Duquette, Jan Novakofski, Nohra E. Mateus-Pinilla

**Affiliations:** 1 Illinois Natural History Survey-Prairie Research Institute, University of Illinois Urbana-Champaign, Champaign, IL 61801, USA; hedmanh@illinois.edu (H.D.H.); jnova@illinois.edu (J.N.); 2 Department of Pathobiology, College of Veterinary Medicine, University of Illinois Urbana-Champaign, Urbana, IL 61801, USA; cvarga@illinois.edu; 3 Illinois Department of Natural Resources, Division of Wildlife Resources; Champaign, IL 62702, USA; Jared.Duquette@illinois.gov; 4 Department of Animal Sciences, University of Illinois Urbana-Champaign, Urbana, IL 61801, USA

**Keywords:** food safety, foodborne pathogens, One Health, zoonoses, wildlife disease management

## Abstract

Emerging foodborne pathogens present a threat to public health. It is now recognized that several foodborne pathogens originate from wildlife as demonstrated by recent global disease outbreaks. Zoonotic spillover events are closely related to the ubiquity of parasitic, bacterial, and viral pathogens present within human and animal populations and their surrounding environment. Foodborne diseases have economic and international trade impacts, incentivizing effective wildlife disease management. In North America, there are no food safety standards for handling and consumption of free-ranging game meat. Game meat consumption continues to rise in North America; however, this growing practice could place recreational hunters and game meat consumers at increased risk of foodborne diseases. Recreational hunters should follow effective game meat food hygiene practices from harvest to storage and consumption. Here, we provide a synthesis review that evaluates the ecological and epidemiological drivers of foodborne disease risk in North American hunter populations that are associated with the harvest and consumption of terrestrial mammal game meat. We anticipate this work could serve as a foundation of preventive measures that mitigate foodborne disease transmission between free-ranging mammalian and human populations.

## 1. Introduction

Wildlife can facilitate the environmental spread and foodborne transmission of infectious diseases to human populations. Globally, approximately 43% of emerging human infectious diseases originate in wildlife [1,2]. The risk of foodborne transmission remains high among occupationally exposed populations that routinely handle or process animal products through farming or working at slaughter or processing facilities [3,4]. Livestock, pets, and wild animals have been documented to spread enteric zoonotic pathogens to humans through various transmission pathways, including direct contact with infected animals [5,6], consumption of contaminated animal products [7], consumption of raw contaminated vegetables [8], and drinking water contaminated by wildlife [9].

Awareness and knowledge of exposure mechanisms for foodborne diseases are important preventive measures that ensure the health of recreational hunters who engage in the consumption and handling of game meat products [10]. Therefore, it is critical to follow proper food safety measures to minimize foodborne disease transmission from wild animals to hunters.

Game meat harvested from terrestrial mammals and birds is a lean protein source, energy-rich, and full of macronutrients [11,12]. In comparison to large-scale commercial animal agriculture, free-ranging wildlife game populations may exhibit a lower proportion of microbes with antibiotic resistance [13]. Despite the potential nutritional benefits of game meat, there is still a public health risk associated with the handling and consumption of game meat. Exposures to foodborne pathogens could originate from bacteria (e.g., *Salmonella* spp. [14,15]; *Escherichia coli* [16,17]), protozoa (e.g., *Toxoplasma gondii*) [18,19,20], or parasites (e.g., *Trichinella spiralis*) [21,22]. Similarly, animals can harbor environmental contaminants naturally occurring in the environment or introduced into the environment by human activity. Ingestion of these environmental contaminants can lead to adverse health effects on human populations [23]. Increasing awareness of the risks and prevention methods associated with wildlife contact and game meat consumption helps hunters to take steps toward risk reduction.

Hunter-harvested game meat is not inspected by federal or state agencies in the United States, which can lead to uncertainties in determining the foodborne disease risk related to handling and consumption of game meat [24,25,26]. Furthermore, recreational hunters are a demographic that could be at risk for exposure to foodborne pathogens [27]. As recreational hunting is popular in the United States [28], the risk of foodborne exposure could become a pressing public health issue. Knowledge of food safety practices and risks could serve as preventive measures to reduce foodborne pathogen exposure among hunter populations within North America [24].

Throughout many parts of North America, recreational hunters and wildlife managers are essential for regulating mammalian wildlife populations [29]. For example, to sustain overall community ecology and stability purposes, wildlife managers should use focused removal of ungulates to decrease their impact on biota and food webs [30]. Likewise, removing terrestrial mammals from areas with increased disease infection rates is an important disease management strategy. Within the state of Illinois in the United States, localized target culling of deer following a surveillance effort using recreational hunter-harvested deer has been necessary for limiting the spread of chronic wasting disease (CWD) [31,32]. Similarly, the management of bovine tuberculosis in Michigan has required the collective efforts of focused disease management programs and recreational hunters to reduce pathogen spread between livestock and wildlife populations [33]. These examples demonstrate the value of collaborations between wildlife conservation authorities and recreational hunters in managing wildlife disease spread.

Because of the complexity of disease transmission, there is a need for an integrative One Health framework for managing foodborne disease transmission among animals, humans, and the environment [34,35,36]. Here, we provide a synthesis review that evaluates risk factors of foodborne diseases related to wild terrestrial large-mammalian game meat handling and consumption, with attention towards recreational hunter populations. We share these findings to support recreational hunters in reducing the health burden of foodborne diseases.

## 2. Infectious Disease Transmission Risk from Wildlife Baiting and Shared Environment

The practice of baiting and supplemental feeding is sometimes implemented in recreational game meat harvest and could facilitate opportunities for interspecies disease transmission [37]. Although not always successful, supplemental feeding of game animals is used to alleviate various ecological and economic factors that can include winter mortality [38], wildlife damage to crops [39], wildlife-vehicle collisions [40], wildlife migration [41], and enriching recreational hunting and tourism prospects [42,43]. However, the negative impact of supplementary feeding for free-ranging wildlife remains controversial due to its risk to increase disease transmission, and impact on human and wildlife health [37]. In North America, the practice of supplementary feeding of cervids has contributed to the spread of chronic wasting disease [44,45], tuberculosis [46], and brucellosis in free-ranging and captive cervids [47]. Numerous studies have documented that supplemental feeding can facilitate large concentrations of wildlife foraging near feeding sites, expanding the risk of inter- and intra-species transmission of infectious diseases [48,49]. Feeding sites propagate the transmission of pathogens through direct contact (e.g., muzzle contact, sparring) or indirect transmission via environmental fecal contamination [50]. Practices that promote supplemental feeding of wildlife can enable foodborne disease transmission between domestic and wildlife species.

Free-ranging livestock and poultry can overlap in shared pastures and water sources, increasing the risk of transmission among wildlife species and human populations [51,52,53,54]. Further spatial epidemiological surveillance of animal movement patterns could help identify areas of inter-species transmission [55]. These findings could prove beneficial to better inform hunters of risk areas to avoid harvest from. To effectively study this issue, a One Health approach is necessary to facilitate cross-disciplinary collaborations to monitor foodborne infections at the intersection of animals, humans, and the environment.

## 3. Game Meat Hygiene

**Figure 1:**
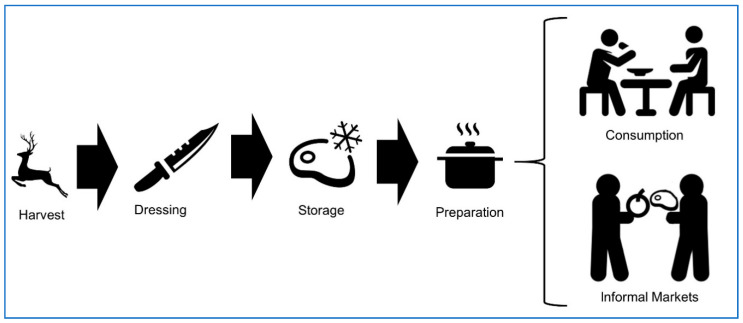
Conceptual diagram of the food chain process of terrestrial mammalian game meat in North America.

Understanding sources and use of game meat in the North American context can help develop a foodborne disease prevention and control system. Game meats are widely defined as animal products harvested from free-ranging, non-domesticated, or captive wildlife [26]. We align our review with North American hunter-harvest, focusing on the consumption and handling of free-ranging terrestrial mammalian game animals. Although hunter food safety is a clearly defined area within public health, we acknowledge there are many important peripheral topics relevant to the practice of game meat harvest including the export and import of game meats, informal and illegal game meat market systems, and captive-reared wildlife [10]. Game meat harvest and consumption in North America differs from commercial food animal production in the following attributes: slaughter, evisceration, storage, inspection, and market distribution [10]. Recreational hunter-harvest within North America consists of (1) harvest, (2) carcass dressing, (3) storage, and (4) informal market trade or consumption (Figure 1). In each of these phases of the game meat chain, there is a risk of exposure to foodborne pathogens. Vulnerable groups, such as the elderly, immunocompromised individuals, pregnant women, and infants are at increased risk of morbidity and mortality from foodborne infections and should avoid eating raw or undercooked seafood, poultry, and meat [56] including game meat.

In contrast to North America, many European nations permit the commercial trade of game animal products [57]. Moreover, the consumption of regulated game meat products continues to grow throughout Europe [57,58]. Member states of the European Union require hunters that participate in the selling of game meat to abide by EC Regulation N.178/2002, which outlines that independent hunters are responsible for demonstrating substantial knowledge of infectious disease symptoms to accurately identify them during initial carcass inspection [59].

In the application of firearms or archery, a quick and precise kill is essential. The quality of the harvest is determined by the target of the bullet, arrow, or bolt. Hunters are encouraged to aim for vital organs and accurately recognize these targets in both small and big game animals [60]. A growing body of evidence suggests that it is important to use lead-free ammunition for a firearm-killed game because lead fragments can persist in animal tissue, leading to human health risks associated with lead ingestion [61,62].

In North America, internal organ removal, especially the intestine is a critical stage in the game meat harvest, where hunters are often working in outdoor settings with limited access to sanitation and hygienic infrastructure. Hunting environments can present similar challenges as in low-resource settings in terms of access to potable water and sanitary processing environments [63,64]. Similarly, the majority of game animals are killed in natural environments where sticking, bleeding, and eviscerations are carried out [65]. Processing of carcass entails three critical steps: (1) field dressing, (2) cutting and processing, and (3) disposal of inedible organs and carcass parts. The Illinois Department of Agriculture provides informative instructions for cervid meat processing that outlines to use rubber or latex gloves and use dedicated knives for game meat processing [66]. The Illinois Department of Agriculture recommends avoiding contact with the hide, brain, spinal cord, spleen, eyes, tonsils, or lymph nodes [66]. It is important to cut through the spinal column only when removing the head and to use a designated knife for this purpose [66]. Careful removal of the anus and intestine is important to reduce fecal contamination of the meat, preventing carcass contamination with enteric pathogens [67]. The hide and skin are kept as protection of the meat against contamination during the evisceration process [67]. Meat should be cooled immediately to reduce bacterial growth and secondarily to uphold the best flavor. Disease transmission from game carcasses to avian scavengers is plausible [68]. Proper disposal of carcass remains is recommended to limit the environmental transmission of pathogens among scavengers and other wildlife [10].

Once the carcass is processed, it is necessary to keep the meat at 1 °C–4 °C [69,70], or frozen to minimize microbial growth. It is recommended that hunters seeking professional processing services ensure freezing units are available [10]. Properly packaged game meat can be stored up to on average 12 months while uncured venison can be stored notably longer [69]. Unfrozen game meats should be stored at 4 °C or less and should be prepared within 2–3 days [71]. Inside of freezer units, game meat should be in labeled and sealed packages with adequate space to separate them from other products. It is recommended to place raw game meat products on bottom shelves to avoid contamination of other products from dripping meat juices [72]. Foodborne infections can be prevented by cooking steaks and ground meats at minimum temperatures of 71 and 74 °C respectively [70,73,74].

In North America, it is not uncommon for hunters to share processed game meat through informal markets that are largely driven by their social networks. In Michigan, most of the hunter (75% [95% CI: 71–78%]) and non-hunter (59% [95% CI: 54–65%]) populations reported consumption of game meat [75]. Meanwhile, Michiganders that have not consumed game meat reported diet and taste as leading factors for never consuming game meat [75]. Factors that had the greatest influence on the frequency of game meat consumption included hunting experience, social network, race, and urbanicity of place of residence [75]. Another study in Michigan documented that deer hunters most commonly shared meat within tight social networks, including household members (69%), relatives (52%), friends neighbors, or coworkers (50%) [76].

The final use of the game meat through consumption or informal market trade can impact the spread of potential pathogens not only to hunter populations but also to those within their social networks [77,78]. Game meat throughout the world is presumed to be attained through localized market networks [77]. Although commerce of game meat in North America is illegal, it is not uncommon for recreational hunters to engage in informal markets or freely donate game meat to individuals closely linked to them within their social networks or to meat processors who provide the meat to food banks that distribute meat to the public [75,76,78]. In Illinois, venison from hunter-harvested deer and deer from the Illinois Department of Natural Resources (IDNR) managed local targeted culling CWD program is donated to food banks. Deer from the IDNR are tested for CWD before being donated and processed to avoid CWD positive deer from entering the food chain. However, hunter-harvested deer may not be tested for CWD in some cases.

Local meat consumption prevents regional outbreaks of foodborne infections that are more likely to occur through commercial food supply chains [79,80]. In the end, it is imperative that hunters carefully inform persons that they share game meat products with the origins and conditions of the game meat before consumption.

## 4. Overview of Foodborne Diseases

### 4.1. Bacterial Pathogens

Deficient hunter-harvest food safety practices increase the transmission risk of bacterial pathogens. Some of the most prevalent bacterial foodborne pathogens related to inadequate practices include *Escherichia coli*, *Salmonella* spp., *Campylobacter* spp., *Yersinia enterocolitica*, *Listeria monocytogenes*, and *Leptospira interrogans* [17,81,82,83].

In North America, several bacterial foodborne disease outbreaks have been associated with the consumption of terrestrial game meat. In Oregon, United States of America (USA), an *Escherichia coli* 0157:H7 community outbreak was related to the consumption of homemade venison jerky [17]. In Connecticut, USA the consumption of undercooked grilled venison tenderloin of white-tailed deer was connected to a severe gastrointestinal *Escherichia coli* O157:H7 infection in a young boy [84]. In Oregon, USA, consumption of locally grown fresh strawberries contaminated with black-tailed deer feces was associated with an *Escherichia coli* O157:H7 outbreak [85]. In the Hawaiian island of Lana’I, USA, *Salmonella* Birkenhead infections were related to the consumption of raw venison sashimi made from axis deer [86] (Table 1).

Only a few studies have specifically analyzed the impacts of hunter food safety procedures on the contamination of deer and moose carcasses [10,87,88,89]. Bacterial foodborne diseases are more common during the summer [90,91,92]. For example, in Europe, higher bacterial colony counts have been detected on game meat carcasses during the summer compared to the winter season [93]. Often hunters submit their game meat to a slaughter plant for processing. Game meat processing plants can be variable in the quality of sanitation methods they utilize [24]. In the absence of standardized food safety regulations, the risk of microbial contamination of carcasses might differ among various regions [89,94].

There are no reports of outbreaks in North America in humans of *Mycobacterium bovis* (Bovine tuberculosis), *Brucella suis*, or *Brucella abortus* associated with terrestrial game meat consumption. However, hunters’ exposure to these zoonotic pathogens in cervids [95,96], feral swine [97], and bison and elk [98] may occur while field-dressing infected animals.

### 4.2. Parasites

Parasitic foodborne diseases represent a diverse group of pathogens [99]. Parasitic human water- and food-borne infections can occur indirectly through ingestion of water contaminated by game mammals (e.g., cryptosporidiosis, giardiasis) [100] or directly through the ingestion of game meat products infected with the cyst stage of the parasite (e.g., *Trichinella* spp.) [101]. These parasites can persist in terrestrial wildlife [102], remaining infective in their muscular tissues. Therefore, preventive measures for select parasites do not always apply to others. For example, meat inspection is the foremost food safety measure to manage *Trichinella* spp. in domestic animals, but this practice is rarely done for terrestrial game animals [103,104] and not conducted by the state or federal agencies in the United States [24,70]. However, pathogens like *Toxoplasma gondii* where the game animal can serve as an intermediate host containing the cyst stage of the parasite [105], may go unnoticed during meat inspection [24,105,106]. Another underlying concern is the frequent contamination of soil and water reservoirs where parasites can persist for long periods until they infect the subsequent hosts in their life cycle [107,108]. These varying risk factors in tandem with an already severely under-recognized field, complicate effective parasitic disease prevention, and surveillance among free-ranging terrestrial mammal populations.

In North America, many foodborne parasitic diseases related to terrestrial mammal game meat consumption have been described previously. Consuming undercooked black bear meat was linked to *Trichinella nativa* infections in Saskatchewan, Canada [109], and *Trichinella murrelli* infections in Illinois, USA [110]. Moreover, an outbreak of acute toxoplasmosis among Canadian hunters were linked to the consumption of undercooked white-tailed deer harvested in Illinois, USA [20] (Table 1).

### 4.3. Viruses

Hepatitis E, an emerging foodborne viral pathogen, has been previously described as a risk for humans who consume game meat. Besides, rabies and *Parapoxvirus* infections are important zoonotic diseases that hunters can acquire through direct contact with infected game animals.

According to the Centers for Disease Control (CDC), in 2018, rabies cases of wildlife accounted for 92.7% of total reported cases in the United States [111]. Bats comprised the most frequent rabid animals (33%), followed by raccoons (30.3%), skunks (20.3%), and foxes (7.2%). Hunters and their hunting dogs might be at risk of contracting rabies through direct contact with infected wildlife. Furthermore, as the rabies virus can infect any mammal, contracting rabies through direct contact with infected game animal carcasses such as deer or bear cannot be excluded [112,113].

While not documented in North America, previous Japanese studies described human cases of hepatitis E virus linked to the consumption of raw or undercooked wild boar [114,115], and deer meat [116]. Finally, in the United States, *Parapoxvirus* infections have occurred through direct contact with infected deer carcasses [117,118,119].

### 4.4. Lead Exposure

Lead (Pb) is a highly neurotoxic and persistent element [120]. In the United States, hunting and shooting firearms yield the greatest discharges of unregulated lead into the environment [121]. To date, several European countries including Denmark, the Netherlands, and Sweden have instituted complete bans on the use of lead ammunition [122]. Support remains a challenge for nation-wide bans in North America [123]. Game meat can contain variable concentrations of lead in the form of residues from hunting ammunition [61]. Lead particulates do not necessarily surmount an immediate health risk [124]. Frequent ingestion of game meat did not have a significant impact on hunter blood lead levels [125]. Prolonged ingestion of lead sources might result in toxicity [61,126,127]. This supports the idea that many aspects of meat contamination are understudied, particularly concerning their potential impact on humans. Previous research articles have identified a negative impact of lead on wildlife health [120,128,129]. Unregulated use of lead ammunition presents a health exposure risk to at least 10 million recreation hunters, their families, and close contacts, and beneficiaries from game meat donations [61].

## 5. Conclusions

Hunting has been part of cultural practices for millennia. However, the popularity of recreational hunting in North America coupled with limited awareness of food safety preventive measures might pose a foodborne infection risk to hunters, their families, and the broader community who consume game meat as a gift or donation from food banks.

Recreational hunting is a practice that will likely remain popular, and there is a need for education and extension programs to disseminate food safety guidelines and best practices. Using a One Health approach by recognizing connections among animals, humans and their shared environments will benefit hunters in preventing foodborne diseases.

In North America, currently, there are no food safety standards for game meat harvested for personal consumption. Therefore, we outlined prevention opportunities to mitigate hunters’ and game meat consumers’ risk of contracting zoonotic foodborne pathogens.

## Figures and Tables

**Table 1:** Overview of the primary foodborne diseases originating from free-ranging mammal game animals in North America.

Geographic Region	Pathogen	Species	Transmission Pathway	Reference
Oregon, USA	*Escherichia coli* 0157:H7	Black-tailed deer (*Odocoileus hemionus*)	Communal consumption of homemade venison jerky	[17]
Connecticut, USA	*Escherichia coli* 0157:H7	White-tailed deer (*Odocoileus virginianus*)	Consumption of undercooked meat	[84]
Oregon, USA	*Escherichia coli* 0157:H7	Black-tailed deer (*Odocoileus hemionus*)	Consumption of strawberries contaminated by deer feces	[85]
Hawaiian island of Lana’I, USA	*Salmonella* Birkenhead	Axis deer (*Axis axis*)	Consumption of undercooked meat	[86]
Illinois, USA	*Toxoplasma gondii*	White-tailed deer (*Odocoileus virginianus*)	Consumption of undercooked meat	[20]
Saskatchewan, CAN	*Trichinella nativa*	Black bear (*Ursus americanus*)	Consumption of undercooked meat	[109]
California, USA	*Trichinella murrelli*	Black bear (*Ursus americanus*)	Consumption of undercooked meat	[110]

## References

[1] Jones K.E., Patel N.G., Levy M.A., Storeygard A., Balk D., Gittleman J.L., Daszak P. (2008). Global trends in emerging infectious diseases. Nature.

[2] Watsa M. (2020). Rigorous wildlife disease surveillance. Science.

[3] Su C., Stover D.T., Buss B.F., Carlson A.V. (2017). LS Occupational animal exposure among persons with campylobacteriosis and cryptosporidiosis—Nebraska, 2005–2015. MMWR Morb. Mortal. Wkly. Rep..

[4] Su C., de Perio M.A., Fagan K., Smith M.L., Salehi E., Levine S., Gruszynski K., Luckhaupt S.E. (2017). Occupational distribution of campylobacteriosis and salmonellosis cases—Maryland, Ohio, and Virginia, 2014. MMWR. Morb. Mortal. Wkly. Rep..

[5] Conrad C.C., Stanford K., Narvaez-Bravo C., Callaway T., McAllister T. (2017). Farm fairs and petting zoos: A review of animal contact as a source of zoonotic enteric disease. Foodborne Pathog. Dis..

[6] Varga C., Middleton D., Walton R., Savage R., Tighe M.-K., Allen V., Ahmed R., Rosella L. (2012). Evaluating risk factors for endemic human *Salmonella* Enteritidis infections with different phage types in Ontario, Canada using multinomial logistic regression and a case-case study approach. BMC Public Health.

[7] Li M., Havelaar A.H., Hoffmann S., Hald T., Kirk M.D., Torgerson P.R., Devleesschauwer B. (2019). Global disease burden of pathogens in animal source foods, 2010. PLoS ONE.

[8] Bennett S.D., Sodha S.V., Ayers T.L., Lynch M.F., Gould L.H., Tauxe R.V. (2018). Produce-associated foodborne disease outbreaks, USA, 1998–2013. Epidemiol. Infect..

[9] Zahedi A., Paparini A., Jian F., Robertson I., Ryan U. (2016). Public health significance of zoonotic *Cryptosporidium* species in wildlife: Critical insights into better drinking water management. Int. J. Parasitol. Parasites Wildl..

[10] Paulsen P., Bauer A., Vodnansky M., Winkelmayer R., Smulders F.J.M., Paulsen P., Bauer A. (2011). Game Meat Hygiene in Focus Microbiology, Epidemiology, Risk Analysis and Quality Assurance.

[11] Hoffman L.C., Wiklund E. (2006). Game and venison—meat for the modern consumer. Meat Sci..

[12] Valencak T.G., Gamsjäger L., Ohrnberger S., Culbert N.J., Ruf T. (2015). Healthy n-6/n-3 fatty acid composition from five European game meat species remains after cooking. BMC Res. Notes.

[13] Lillehaug A., Bergsjø B., Schau J., Bruheim T., Vikøren T., Handeland K. (2005). *Campylobacter* spp., *Salmonella* spp., verocytotoxic *Escherichia coli*, and antibiotic resistance in indicator organisms in wild cervids. Acta Vet. Scand..

[14] Vieira-Pinto M., Morais L., Caleja C., Themudo P., Torres C., Igrejas G., Poeta P., Martins C. (2011). *Salmonella* sp. in game (*Sus scrofa* and *Oryctolagus cuniculus*). Foodborne Pathog. Dis..

[15] Hilbert F., Smulders F.J.M., Chopra-Dewasthaly R., Paulsen P. (2012). *Salmonella* in the wildlife-human interface. Food Res. Int..

[16] Miko A., Pries K., Haby S., Steege K., Albrecht N., Krause G., Beutin L. (2009). Assessment of Shiga toxin-producing *Escherichia coli* isolates from wildlife meat as potential pathogens for humans. Appl. Environ. Microbiol..

[17] Keene W.E. (1997). An outbreak of *Escherichia coli* 0157:H7 infections traced to jerky made from deer meat. JAMA J. Am. Med. Assoc..

[18] Hove T., Mukaratirwa S. (2005). Seroprevalence of *Toxoplasma gondii* in farm-reared ostriches and wild game species from Zimbabwe. Acta Trop..

[19] Malmsten J., Jakubek E.-B., Björkman C. (2011). Prevalence of antibodies against *Toxoplasma gondii* and *Neospora caninum* in moose (Alces alces) and roe deer (Capreolus capreolus) in Sweden. Vet. Parasitol..

[20] Gaulin C., Ramsay D., Thivierge K., Tataryn J., Courville A., Martin C., Cunningham P., Désilets J., Morin D., Dion R. (2020). Acute toxoplasmosis among Canadian deer hunters associated with consumption of undercooked deer meat hunted in the United States. Emerg. Infect. Dis..

[21] Murrell K.D. (2016). The dynamics of *Trichinella spiralis* epidemiology: Out to pasture?. Vet. Parasitol..

[22] Holzbauer S.M., Agger W.A., Hall R.L., Johnson G.M., Schmitt D., Garvey A., Bishop H.S., Rivera H., de Almeida M.E., Hill D. (2014). Outbreak of *Trichinella spiralis* infections associated with a wild boar hunted at a game farm in Iowa. Clin. Infect. Dis..

[23] Warenik-Bany M., Maszewski S., Mikolajczyk S., Piskorska-Pliszczynska J. (2019). Impact of environmental pollution on PCDD/F and PCB bioaccumulation in game animals. Environ. Pollut..

[24] American Veterinary Medical Association (AVMA) Disease Precautions for Hunters. https://www.avma.org/resources/public-health/disease-precautions-hunters.

[25] United States Department of Agriculture (USDA) Roasting Those “Other” Holiday Meats. https://www.fsis.usda.gov/wps/portal/fsis/topics/food-safety-education/get-answers/food-safety-fact-sheets/seasonal-food-safety/roasting-those-other-holiday-meats/ct_index.

[26] United States Department of Agriculture (USDA) Harvesting Wild Game. https://www.fsis.usda.gov/wps/wcm/connect/fsis-content/internet/main/newsroom/meetings/newsletters/small-plant-news/small-plant-news-archive/volume-5/spn-vol5-no4.

[27] Schantz P.M., Moorhead A., Grunenwald P.E., Dietz V.J. (1999). Trichinellosis in the United States, 1991–1996: Declining but not gone. Am. J. Trop. Med. Hyg..

[28] U.S. Department of the Interior, U.S. Fish and Wildlife Service, U.S. Department of Commerce, U.S. Census Bureau 2011 National Survey of Fishing, Hunting, and Wildlife-Associated Recreation.

[29] Gamborg C., Sandøe P., Palmer C. (2020). Ethical management of wildlife. Lethal versus nonlethal control of white-tailed deer. Conserv. Sci. Pract..

[30] Blossey B., Curtis P., Boulanger J., Dávalos A. (2019). Red oak seedlings as indicators of deer browse pressure: Gauging the outcome of different white-tailed deer management approaches. Ecol. Evol..

[31] Mateus-Pinilla N., Weng H.-Y., Ruiz M.O., Shelton P., Novakofski J. (2013). Evaluation of a wild white-tailed deer population management program for controlling chronic wasting disease in Illinois, 2003–2008. Prev. Vet. Med..

[32] Manjerovic M.B., Green M.L., Mateus-Pinilla N., Novakofski J. (2014). The importance of localized culling in stabilizing chronic wasting disease prevalence in white-tailed deer populations. Prev. Vet. Med..

[33] VerCauteren K.C., Lavelle M.J., Campa H. (2018). Persistent spillback of bovine tuberculosis from white-tailed deer to cattle in Michigan, USA: Status, strategies, and needs. Front. Vet. Sci..

[34] Cunningham A.A., Daszak P., Wood J.L.N. (2017). One Health, emerging infectious diseases and wildlife: Two decades of progress?. Philos. Trans. R. Soc. B.

[35] Jenkins E.J., Simon A., Bachand N., Stephen C. (2015). Wildlife parasites in a One Health world. Trends Parasitol..

[36] Godfroid J. (2017). Brucellosis in livestock and wildlife: Zoonotic diseases without pandemic potential in need of innovative one health approaches. Arch. Public Health.

[37] Sorensen A., van Beest F.M., Brook R.K. (2014). Impacts of wildlife baiting and supplemental feeding on infectious disease transmission risk: A synthesis of knowledge. Prev. Vet. Med..

[38] Weidman T., Litvaitis J.A. (2011). Can supplemental food increase winter survival of a threatened cottontail rabbit?. Biol. Conserv..

[39] Mathisen K.M., Milner J.M., van Beest F.M., Skarpe C. (2014). Long-term effects of supplementary feeding of moose on browsing impact at a landscape scale. For. Ecol. Manag..

[40] Andreassen H.P., Gundersen H., Storaas T. (2005). The effect of scent-marking, forest clearing, and supplemental feeding on moose-train collisions. J. Wildl. Manag..

[41] Sahlsten J., Bunnefeld N., Månsson J., Ericsson G., Bergström R., Dettki H. (2010). Can supplementary feeding be used to redistribute moose Alces alces?. Wildl. Biol..

[42] Geisser H., Reyner H.U. (2004). Efficacy of hunting, feeding, and fencing to reduce crop damage by wild boars. J. Wildl. Dis..

[43] Steyaert S.M.J.G., Kindberg J., Jerina K., Krofel M., Stergar M., Swenson J.E., Zedrosser A. (2014). Behavioral correlates of supplementary feeding of wildlife: Can general conclusions be drawn?. Basic Appl. Ecol..

[44] Mysterud A., Viljugrein H., Solberg E.J., Rolandsen C.M. (2019). Legal regulation of supplementary cervid feeding facing chronic wasting disease. J. Wildl. Manag..

[45] Thompson A.K., Samuel M.D., Van Deelen T.R. (2008). Alternative feeding strategies and potential disease transmission in Wisconsin white-tailed deer. J. Wildl. Manag..

[46] Zanella G., Duvauchelle A., Hars J., Moutou F., Boschiroli M.L., Durand B. (2008). Patterns of lesions of bovine tuberculosis in wild red deer and wild boar. Vet. Rec..

[47] Putman R.J., Staines B.W. (2004). Supplementary winter feeding of wild red deer Cervus elaphus in Europe and North America: Justifications, feeding practice and effectiveness. Mamm. Rev..

[48] Robb G.N., McDonald R.A., Chamberlain D.E., Bearhop S. (2008). Food for thought: Supplementary feeding as a driver of ecological change in avian populations. Front. Ecol. Environ..

[49] Murray M.H., Becker D.J., Hall R.J., Hernandez S.M. (2016). Wildlife health and supplemental feeding: A review and management recommendations. Biol. Conserv..

[50] Lavelle M.J., Phillips G.E., Fischer J.W., Burke P.W., Seward N.W., Stahl R.S., Nichols T.A., Wunder B.A., VerCauteren K.C. (2014). Mineral licks: Motivational factors for visitation and accompanying disease risk at communal use sites of elk and deer. Environ. Geochem. Health.

[51] Craft M.E. (2015). Infectious disease transmission and contact networks in wildlife and livestock. Philos. Trans. R. Soc. B Biol. Sci..

[52] Daniels M.J., Hutchings M.R., Greig A. (2003). The risk of disease transmission to livestock posed by contamination of farm stored feed by wildlife excreta. Epidemiol. Infect..

[53] Hedman H.D., Zhang L., Trueba G., Vinueza Rivera D.L., Zurita Herrera R.A., Villacis Barrazueta J.J., Gavilanes Rodriguez G.I., Butt B., Foufopoulos J., Berrocal V.J. (2020). Spatial exposure of agricultural antimicrobial resistance in relation to free-ranging domestic chicken movement patterns among agricultural communities in Ecuador. Am. J. Trop. Med. Hyg..

[54] Brochu N.M., Guerin M.T., Varga C., Lillie B.N., Brash M.L., Susta L. (2019). A two-year prospective study of small poultry flocks in Ontario, Canada, part 1: Prevalence of viral and bacterial pathogens. J. Vet. Diagn. Investig..

[55] Thomas L.F., de Glanville W.A., Cook E.A., Fevre E.M. (2013). The spatial ecology of free-ranging domestic pigs (Sus scrofa) in western Kenya. BMC Vet. Res..

[56] Lund B.M., O’Brien S.J. (2011). The occurrence and prevention of foodborne disease in vulnerable people. Foodborne Pathog. Dis..

[57] Giuggioli G., Olivastri A., Pennisi L., Paludi D., Ianieri A., Vergara A. (2018). The hygiene-sanitary control in the wild game meats. Ital. J. Food Saf..

[58] Mesinger D., Ocieczek A. (2020). Consumer education as an important condition for increasing wild animal meat consumption in the context of promoting the idea of sustainable development in Poland. Pol. J. Environ. Stud..

[59] European Parliament and of the Council Regulation (EC) No 178/2002 of the European Parliament and of the Council of 28 January 2002 Laying Down the General Principles and Requirements of Food Law, Establishing the European Food Safety Authority and Laying Down Procedures in Matters of Food Safty. https://eur-lex.europa.eu/legal-content/EN/ALL/?uri=celex%3A32002R0178.

[60] Illinois Department of Natural Resources (IDNR) Summary of 2013–2014 Illinois Deer Seasons. https://www2.illinois.gov/dnr/hunting/deer/Documents/2019-2020IllinoisDeerHarvestReport.pdf.

[61] Hunt W.G., Watson R.T., Oaks J.L., Parish C.N., Burnham K.K., Tucker R.L., Belthoff J.R., Hart G. (2009). Lead bullet fragments in venison from rifle-killed deer: Potential for human dietary exposure. PLoS ONE.

[62] Buenz E.J., Parry G.J. (2018). Chronic lead intoxication from eating wild-harvested game. Am. J. Med..

[63] Prüss-Ustün A., Bartram J., Clasen T., Colford J.M., Cumming O., Curtis V., Bonjour S., Dangour A.D., De France J., Fewtrell L. (2014). Burden of disease from inadequate water, sanitation and hygiene in low- and middle-income settings: A retrospective analysis of data from 145 countries. Trop. Med. Int. Health.

[64] Arnold B.F., Null C., Luby S.P., Unicomb L., Stewart C.P., Dewey K.G., Ahmed T., Ashraf S., Christensen G., Clasen T. (2013). Cluster-randomised controlled trials of individual and combined water, sanitation, hygiene and nutritional interventions in rural Bangladesh and Kenya: The WASH Benefits study design and rationale. BMJ Open.

[65] Gill C.O. (2007). Microbiological conditions of meats from large game animals and birds. Meat Sci..

[66] Illinois Department of Agriculture (IDOA) Handling Carcasses and Venison. https://www2.illinois.gov/sites/agr/Animals/AnimalHealth/AnimalDiseases/Pages/CWD-Processing.aspx.

[67] Paulsen P., Smulders F.J.M., Hilbert F. (2012). *Salmonella* in meat from hunted game: A central European perspective. Food Res. Int..

[68] Carrasco-Garcia R., Barroso P., Perez-Olivares J., Montoro V., Vicente J. (2018). Consumption of big game remains by scavengers: A potential risk as regards disease transmission in central Spain. Front. Vet. Sci..

[69] United States Department of Agriculture (USDA) FSIS Compliance Guide: Modernization of Poultry Slaughter. https://www.fsis.usda.gov/wps/wcm/connect/7a0a728e-3b29-49e9-9c1b-ec55f2f04887/Chilling-Requirements-1014.pdf?MOD=AJPERES.

[70] United States Department of Agriculture (USDA) Keep Food Safe! Food Safety Basics. https://www.fsis.usda.gov/wps/portal/fsis/topics/food-safety-education/get-answers/food-safety-fact-sheets/safe-food-handling/keep-food-safe-food-safety-basics/CT_Index/!ut/p/a1/jZHRToMwFIafhsvSInNh3hESM9CBy9R13CwFDqURWkKrqE9vtyXGmU3XXvWc78tp_-IcU5xL9iY4M.

[71] University of Minnesota Extension Cooking Venison for Flavor and Safety. https://extension.umn.edu/preserving-and-preparing/cooking-venison-flavor-and-safety.

[72] Clemson Cooperative Extension Safe handling of Wild Game Meats. Factsheet.HGIC 3516. https://hgic.clemson.edu/factsheet/safe-handling-of-wild-game-meats.

[73] United States Department of Agriculture (USDA) Foodborne Illness Peaks in Summer—What Can You Do to Prevent It?. https://www.fsis.usda.gov/wps/portal/fsis/topics/food-safety-education/get-answers/food-safety-fact-sheets/foodborne-illness-and-disease/foodborne-illness-peaks-in-summer/ct_index#:~:text=Cookmeatandpoultrycompletely,°F%2F74°C.

[74] Bryan F.L. (1978). Factors that contribute to outbreaks of foodborne disease. J. Food Prot..

[75] Goguen A.D., Riley S.J. (2020). Consumption of wild-harvested meat in society. Wildl. Soc. Bull..

[76] Goguen A.D., Riley S.J., Organ J.F., Rudolph B.A. (2018). Wild-harvested venison yields and sharing by Michigan deer hunters. Hum. Dimens. Wildl..

[77] Ljung P.E., Riley S.J., Heberlein T.A., Ericsson G. (2012). Eat prey and love: Game-meat consumption and attitudes toward hunting. Wildl. Soc. Bull..

[78] Illinois Department of Natural Resources (IDNR) Chronic Wasting Disease Management. https://www2.illinois.gov/dnr/programs/CWD/Pages/default.aspx.

[79] Dorn C.R. (1993). Review of foodborne outbreak of *Escherichia coli* O157:H7 infection in the western United States. J. Am. Vet. Med. Assoc..

[80] Bell B.P. (1994). A Multistate outbreak of *Escherichia coli* O157:H7—Associated bloody diarrhea and hemolytic uremic syndrome from hamburgers. JAMA J. Am. Med. Assoc..

[81] Meng X.J., Lindsay D.S., Sriranganathan N. (2009). Wild boars as sources for infectious diseases in livestock and humans. Philos. Trans. R. Soc. B Biol. Sci..

[82] Chlebicz A., Śliżewska K. (2018). Campylobacteriosis, salmonellosis, yersiniosis, and listeriosis as zoonotic foodborne diseases: A review. Int. J. Environ. Res. Public Health.

[83] Membré J.-M., Laroche M., Magras C. (2011). Assessment of levels of bacterial contamination of large wild game meat in Europe. Food Microbiol..

[84] Rabatsky-Ehr T., Dingman D., Marcus R., Howard R., Kinney A., Mshar P. (2002). Deer meat as the source for a sporadic case of *Escherichia coli* O157:H7 infection, Connecticut. Emerg. Infect. Dis..

[85] Laidler M.R., Tourdjman M., Buser G.L., Hostetler T., Repp K.K., Leman R., Samadpour M., Keene W.E. (2013). *Escherichia coli* O157:H7 infections associated with consumption of locally grown strawberries contaminated by deer. Clin. Infect. Dis..

[86] Madar C.S., Cardile A.P., Cunningham S., Magpantay G., Finger D. (2012). A case of *Salmonella* gastroenteritis following ingestion of raw venison sashimi. Hawaii J. Med. Public Health.

[87] Atanassova V., Apelt J., Reich F., Klein G. (2008). Microbiological quality of freshly shot game in Germany. Meat Sci..

[88] Avagnina A., Nucera D., Grassi M.A., Ferroglio E., Dalmasso A., Civera T. (2012). The microbiological conditions of carcasses from large game animals in Italy. Meat Sci..

[89] Obwegeser T., Stephan R., Hofer E., Zweifel C. (2012). Shedding of foodborne pathogens and microbial carcass contamination of hunted wild ruminants. Vet. Microbiol..

[90] Lin S., Sun M., Fitzgerald E., Hwang S.-A. (2016). Did summer weather factors affect gastrointestinal infection hospitalizations in New York State?. Sci. Total Environ..

[91] Wallace D.J., Van Gilder T., Shallow S., Fiorentino T., Segler S.D., Smith K.E., Shiferaw B., Etzel R., Garthright W.E., Angulo F.J. (2000). Incidence of foodborne illnesses reported by the Foodborne Diseases Active Surveillance Network (FoodNet)—1997. J. Food Prot..

[92] Varga C., John P., Cooke M., Majowicz S.E. (2020). Spatial and space-time clustering and demographic characteristics of human nontyphoidal *Salmonella* infections with major serotypes in Toronto, Canada. PLoS ONE.

[93] Paulsen P., Winkelmayer R. (2004). Seasonal variation in the microbial contamination of game carcasses in an Austrian hunting area. Eur. J. Wildl. Res..

[94] Sauvala M., Laaksonen S., Laukkanen-Ninios R., Jalava K., Stephan R., Fredriksson-Ahomaa M. (2019). Microbial contamination of moose (Alces alces) and white-tailed deer (Odocoileus virginianus) carcasses harvested by hunters. Food Microbiol..

[95] Sunstrum J., Shoyinka A., Power L.E., Maxwell D., Stobierski M.G., Signs K., Sidge J.L., O’Brien D.J., Robbe-Austerman S., Davidson P. (2019). Notes from the Field: Zoonotic Mycobacterium bovis Disease in Deer Hunters—Michigan, 2002–2017. MMWR Morb. Mortal. Wkly. Rep..

[96] Wilkins M.J., Meyerson J., Bartlett P.C., Spieldenner S.L., Berry D.E., Mosher L.B., Kaneene J.B., Robinson-Dunn B., Stobierski M.G., Boulton M.L. (2008). Human Mycobacterium bovis infection and bovine tuberculosis outbreak, Michigan, 1994–2007. Emerg. Infect. Dis..

[97] Brown V.R., Bowen R.A., Bosco-Lauth A.M. (2018). Zoonotic pathogens from feral swine that pose a significant threat to public health. Transbound. Emerg. Dis..

[98] Olsen S.C. (2010). Brucellosis in the United States: Role and significance of wildlife reservoirs. Vaccine.

[99] Dorny P., Praet N., Deckers N., Gabriel S. (2009). Emerging food-borne parasites. Vet. Parasitol..

[100] Appelbee A.J., Thompson R.C.A., Olson M.E. (2005). *Giardia* and *Cryptosporidium* in mammalian wildlife—Current status and future needs. Trends Parasitol..

[101] Sandfoss M., DePerno C., Patton S., Flowers J., Kennedy-Stoskopf S. (2011). Prevalence of antibody to *toxoplasma gondii* and *trichinella* spp. In feral pigs (Sus scrofa) of eastern North Carolina. J. Wildl. Dis..

[102] Fredebaugh S.L., Mateus-Pinilla N.E., McAllister M., Warner R.E., Weng H.-Y. (2011). Prevalence of antibody to *toxoplasma gondii* in terrestrial wildlife in a natural area. J. Wildl. Dis..

[103] Diaz J.H., Warren R.J., Oster M.J. (2020). The disease ecology, epidemiology, clinical manifestations, and management of trichinellosis linked to consumption of wild animal meat. Wilderness Environ. Med..

[104] Springer Y.P., Casillas S., Helfrich K., Mocan D., Smith M., Arriaga G., Mixson L., Castrodale L., McLaughlin J. (2017). Two outbreaks of trichinellosis linked to consumption of walrus meat—Alaska, 2016–2017. MMWR Morb. Mortal. Wkly. Rep..

[105] Dubey J.P., Brown J., Verma S.K., Cerqueira-Cézar C.K., Banfield J., Kwok O.C.H., Ying Y., Murata F.H.A., Pradhan A.K., Su C. (2017). Isolation of viable *Toxoplasma gondii*, molecular characterization, and seroprevalence in elk (Cervus canadensis) in Pennsylvania, USA. Vet. Parasitol..

[106] Kamerkar S., Davis P.H. (2012). Toxoplasma on the brain: Understanding host-pathogen interactions in chronic CNS infection. J. Parasitol. Res..

[107] Mosites E., Miernyk K., Priest J.W., Bruden D., Hurlburt D., Parkinson A., Klejka J., Hennessy T., Bruce M.G. (2018). *Giardia* and *Cryptosporidium* antibody prevalence and correlates of exposure among Alaska residents, 2007–2008. Epidemiol. Infect..

[108] Shapiro K., Bahia-Oliveira L., Dixon B., Dumètre A., de Wit L.A., VanWormer E., Villena I. (2019). Environmental transmission of *Toxoplasma gondii*: Oocysts in water, soil and food. Food Waterborne Parasitol..

[109] Schellenberg R.S., Tan B.J.K., Irvine J.D., Stockdale D.R., Gajadhar A.A., Serhir B., Botha J., Armstrong C.A., Woods S.A., Blondeau J.M. (2003). An outbreak of trichinellosis due to consumption of bear meat infected with *Trichinella nativa*, in 2 northern Saskatchewan communities. J. Infect. Dis..

[110] Hall R.L., Lindsay A., Hammond C., Montgomery S.P., Wilkins P.P., da Silva A.J., McAuliffe I., de Almeida M., Bishop H., Mathison B. (2012). Outbreak of human trichinellosis in Northern California caused by *Trichinella murrelli*. Am. J. Trop Med. Hyg..

[111] Centers for Disease Control (CDC) Rabies. https://www.cdc.gov/rabies/location/usa/surveillance/wild_animals.html.

[112] Velasco-Villa A., Reeder S.A., Orciari L.A., Yager P.A., Franka R., Blanton J.D., Zuckero L., Hunt P., Oertli E.H., Robinson L.E. (2008). Enzootic rabies elimination from dogs and reemergence in wild terrestrial carnivores, United States. Emerg. Infect. Dis..

[113] Wandeler A.I., Matter H.C., Kappeler A., Budde A. (1993). The ecology of dogs and canine rabies: A selective review. Rev. Sci. Tech..

[114] Yazaki Y., Mizuo H., Takahashi M., Nishizawa T., Sasaki N., Gotanda Y., Okamoto H. (2003). Sporadic acute or fulminant hepatitis E in Hokkaido, Japan, may be food-borne, as suggested by the presence of hepatitis E virus in pig liver as food. J. Gen. Virol..

[115] Matsuda H., Okada K., Takahashi K., Mishiro S. (2003). Severe hepatitis E virus infection after ingestion of uncooked liver from a wild boar. J. Infect. Dis..

[116] Tei S., Kitajima N., Takahashi K., Mishiro S. (2003). Zoonotic transmission of hepatitis E virus from deer to human beings. Lancet.

[117] Roess A.A., McCollum A.M., Gruszynski K., Zhao H., Davidson W., Lafon N., Engelmeyer T., Moyer B., Godfrey C., Kilpatrick H. (2013). Surveillance of parapoxvirus among ruminants in Virginia and Connecticut. Zoonoses Public Health.

[118] Smith K.J., Skelton H.G., James W.D., Lupton G.P. (1991). Parapoxvirus infections acquired after exposure to wildlife. Arch. Dermatol..

[119] Huerter C.J., Hashish H. (2003). A case of human orf contracted from a deer. Cutis.

[120] Pain D.J., Mateo R., Green R.E. (2019). Effects of lead from ammunition on birds and other wildlife: A review and update. AMBIO.

[121] Bannon D.I., Drexler J.W., Fent G.M., Casteel S.W., Hunter P.J., Brattin W.J., Major M.A. (2009). Evaluation of small arms range soils for metal contamination and lead bioavailability. Environ. Sci. Technol..

[122] Mateo R., Kanstrup N. (2019). Regulations on lead ammunition adopted in Europe and evidence of compliance. Ambio.

[123] Arnemo J.M., Andersen O., Stokke S., Thomas V.G., Krone O., Pain D.J., Mateo R. (2016). Health and environmental risks from lead-based ammunition: Science versus socio-politics. Ecohealth.

[124] Treble R.G., Thompson T.S. (2002). Elevated blood lead levels resulting from the ingestion of air rifle pellets. J. Anal. Toxicol..

[125] Haldimann M., Baumgartner A., Zimmerli B. (2002). Intake of lead from game meat—A risk to consumers’ health?. Eur. Food Res. Technol..

[126] Iqbal S., Blumenthal W., Kennedy C., Yip F.Y., Pickard S., Flanders W.D., Loringer K., Kruger K., Caldwell K.L., Jean Brown M. (2009). Hunting with lead: Association between blood lead levels and wild game consumption. Environ. Res..

[127] Buenz E.J. (2016). Lead exposure through eating wild game. Am. J. Med..

[128] Hunt W.G., Burnham W., Parish C.N., Burnham K.K., Mutch B.R., Oaks J.L. (2006). Bullet fragments in deer remains: Implications for lead exposure in avian scavengers. Wildl. Soc. Bull..

[129] Fisher I.J., Pain D.J., Thomas V.G. (2006). A review of lead poisoning from ammunition sources in terrestrial birds. Biol. Conserv..

